# The effect of depressive symptoms on the association between radiographic osteoarthritis and knee pain: a cross-sectional study

**DOI:** 10.1186/1471-2474-14-214

**Published:** 2013-07-22

**Authors:** Duarte Pereira, Milton Severo, Henrique Barros, Jaime Branco, Rui A Santos, Elisabete Ramos

**Affiliations:** 1 Department of Clinical Epidemiology, Predictive Medicine and Public Health, University of Porto Medical School, Alameda Prof. Hernâni Monteiro, 4200-319 Porto, Portugal; 2 Public Health Institute, University of Porto, Porto, Portugal; 3 CEDOC, Nova Medical School / Faculdade de Ciências Médicas, Universidade Nova de Lisboa; Rheumatology Department, CHLO,EPE - Hospital Egas Moniz, Lisboa, Portugal; 4 Beatriz Ângelo Hospital, Loures, Portugal

**Keywords:** Knee, Osteoarthritis, Pain, Radiographic OA, Depressive symptoms

## Abstract

**Background:**

The progressive nature of knee osteoarthritis (OA) leads to not only to physical but also to psychosocial decline; this aspect can influence knee pain experience, manifestations and inevitably diagnostic accuracy.

To analyze the role of depressive symptoms on the association between radiographic OA and knee pain, understanding the ability of knee pain symptoms to find out individuals with radiographic OA.

**Methods:**

Data on 663 subjects was obtained by interview using a structured questionnaire on social, demographic, behavioural and clinical data. Painful knee was assessed regarding having pain: ever, in the last year, in the last 6 months and in the last month. Using factor analysis, participants were graded using a knee pain score, with higher scores representing more symptomatology. Depressive symptoms were evaluated with the Beck Depressive Inventory (BDI), and radiographic knee OA was classified using the Kellgren Lawrence (KL) scale; those with KL ≥ 2 were considered as having radiographic OA.

**Results:**

Knee pain was reported by 53.2% of those with radiographic KL ≥ 2 and by 33.2% of those with radiographic KL < 2. The prevalence of depressive symptoms (BDI > 14) was 19.9% among participants with radiographic KL ≥ 2 and 12.6% among those with radiographic KL < 2 (*p* = 0.01). The association of knee pain with radiographic knee OA was higher in higher pain scores and in participants without depressive symptoms. Among participants with BDI ≤ 14 the likelihood ratio to identify patients with radiographic knee OA increased with increased pain scores: 1.02 for score 1; 2.19 for score 2 and 7.34 when participants responded positively to all pain questions (score 3). Among participants with depressive symptoms (BDI > 14) likelihood ratios were 0.51, 1.92, 1.82, respectively. The results were similar for both genders.

**Conclusions:**

Knee pain scores increased ability to identify participants with radiographic KL ≥ 2 in both sexes. However, the presence of depressive symptoms impairs the ability of knee pain complaints to identify patients with radiographic OA.

## Background

Osteoarthritis (OA) is one of the most frequent causes of pain and disability, representing a substantial burden for the individual and for society [1-3]. Since incidence and prevalence increases with age, longer life expectancy will result in an increase in OA in the future [4,5].

Among the most common joint sites affected by OA, the knee is one of the most prevalent [6]. The knee is a weight-bearing joint, essential for function, and frequently associated with more reported pain in OA [6-8]. Accurate diagnosis and timely intervention is essential to minimize the consequences of knee OA and to slow its progress [9].

Knee OA diagnosis is based on radiographic changes and clinical examination [10]. According to recent recommendations, beside the radiographic evaluation, three symptoms (persistent knee pain, limited morning stiffness and reduced function) and three signs (crepitus, restricted movement and bony enlargement) were considered the most useful in the identification of OA patients [11].

Pain is thought to be an important marker of OA and is frequently the primary reason for seeking health care. It is correlated with radiographic symptomatic changes [12], strongly associated with other signs and symptoms and reliably predicts future disability [13]. Additionally, treatment strategies in OA are frequently focussed on pain relief and control [6]. However, there is a high variability in symptoms among individuals with radiographic findings making it difficult to identify patients with OA and to evaluate the progression of the disease among those already identified [11,14].

Several psycho-social determinants may explain differences in how people experience their symptoms [15]. Pain somatization is a frequent manifestation in depressed people and may predispose patients to report pain more often or even to exacerbate it [16]. Depressive symptoms are a common condition in adults and are frequently un-diagnosed [17]. On the other hand, it is also well-known that depression and its manifestations are prevalent among people with OA [18,19]. While chronic pain itself can cause or aggravate depressive symptoms, the impact of existing depressive symptoms on the experience of pain also needs to be explored [20].

Although recently several studies have evaluated the concordance between radiographic findings and knee pain and have found a strong association [10,16,21], it is important to investigate the role of depressive symptoms in this relation. A comprehensive understanding of these factors can improve diagnosis and clinical approach to patients [15,18]. The aim of this study is to analyze the role of depressive symptoms on the association between radiographic OA and knee pain, understanding the ability of knee pain symptoms to find out individuals with radiographic OA.

## Methods

### Data collection

The study was performed using information collected as part of the *EPIPorto* cohort [22]. Briefly, this cohort evaluates non-institutionalized adults, resident in Porto, an urban centre located in northwest Portugal with almost 400,000 inhabitants. Participants were selected by random digit dialling and invited to visit the Department of Clinical Epidemiology, Predictive Medicine and Public Health for an evaluation, which included an interview based on a structured questionnaire on social, demographic, behavioural, clinical data and physical examination. The proportion of participation was 70%.

The local ethics committee of S. João Hospital, a university hospital, approved the study protocol. All participants gave written consent to participate in the study, which was carried out in accordance with the Helsinki Declaration.

As previously reported, data was collected by trained interviewers using structured questionnaires plus a clinical interview and examination by a physician. We evaluated marital status (categorized on two categories: married/civil union and single/divorced/widow), years of education (measured as the number of successfully completed years of formal schooling), occupation (white collar, blue collar and other, including students, unemployed and those who had never had a job) and current occupational status (working, retired and other). We also evaluated as dichotomous variables (yes/no) regular physical activity practice, chronic medication, self-reported diagnosis of depression, knee OA, other chronic diseases and other rheumatic diseases.

Knee pain was evaluated using a set of “yes/no” questions. Firstly, participants were asked if they “ever had knee pain not related with any trauma or injury?” If participants gave a positive answer they were asked to answer (yes/no) to a further three questions: “In the last year did you have more than 3 knee pain episodes?”; “During the last 6 months did knee pain last longer than a week?”; “During the last month did you have knee pain?” To understand if these questions could be used to measure severity of knee pain, factor analysis for dichotomous variables was performed.

For depressive symptoms the Portuguese version of the *Beck Depression Inventory* (BDI) was used [23]. It is composed of 21 items, evaluating symptoms and attitudes, covering emotions, behavioural changes, and somatic symptoms in the previous 2 weeks before the evaluation. The final score ranges from 0 to 63, with higher scores representing more severe depressive symptoms [24] and those who scored higher than 14 were considered to have depressive symptoms.

Body weight was measured to the nearest 0.1 kg using a digital scale (SECA®), and height was measured to the nearest centimeter using a wall-stadiometer (SECA®); then using body mass index (BMI) [weight (kg)/height (m^2^)] we classified participants in three categories (< 25.0 kg/m^2^ underweight or normal; 25.0 to 29.9 kg/m^2^ overweight; ≥30 kg/m^2^ obese) [25].

Weight-bearing radiographs of the knees (standard anterior-posterior and lateral views) were taken and graded according to the *Kellgren Lawrence scale* (KL): Grade 0, none: no visible features of OA; Grade 1, doubtful: questionable osteophytes or questionable joint space narrowing; Grade 2, minimal: definitive small osteophytes, little/mild joint space narrowing; Grade 3, moderate: definitive moderate osteophytes, joint space narrowing of at least 50%; Grade 4, severe: joint space impaired severely, cysts and sclerosis of subchondral bone [26,27]. A subject was considered to have radiographic OA if the KL score at least on one side was greater or equal to two [14]. Radiographs were scored only by one experienced reader that was unaware of the participants’ clinical data. In order to estimate the intra-observer reliability we calculated the Cronbach’s Alpha [28] which was 0.9 and allowed us to assume as *very good* our intra-observer reliability [29].

### Participants

From the 2485 participants of the *EPIPorto* cohort that participated at the baseline evaluation, 1682 were re-evaluated during the follow-up performed between 2005 and 2008. The first 1000 participants were systematically invited. Compared with the 1682 participants evaluated, participants with radiographic evaluation were significantly lower educated [8.79 (5.07) vs. 9.26 (5.45), p < 0.01] and had a lower proportion of females (58.4% vs. 65.3%, p < 0.01). From the first 1000 participants systematically invited to do radiographs, we obtained 907 knee radiographs; from these 13 participants had unreadable or incomplete knee radiographic evaluation and 231 had missing data for BDI.

After the exclusion process, data on 663 participants (371 females and 292 males) were analysed. The overall median (25th–75th percentile) age was 58 (48–67) years and the overall median BDI score was 7(3–12). Depressive symptoms (BDI > 14) were found in 28.5% of the participants and 17.7% presented both knee pain and depressive symptoms. As shown in Table 1, the 337 participants not included in the analysis were younger than those included [56 (47–66) vs. 58 (48–67) (*p* < 0.01)], but no other characteristics were significantly different among the two groups.

**Table 1:** Comparison between included and excluded participants

		**Excluded n =** **337**	**Evaluated n =** **663**	**p-value**
**Age (years) Mean (SD)**		56.5 (14.1)	58.0 (15.2)	<0.01
**Sex n (%)**	Female	197 (58.5)	371 (56.0)	0.45
**Marital status n (%)**	Married/civil union	238 (70.6)	454 (68.5)	0.46
	Single/divorced/widow	99 (29.4)	209 (31.5)	
**Years of education n (%)**	0-4 years	92 (27.3)	202 (30.5)	0.77
	5-9 years	89 (26.4)	172 (25.9)	
	10-12 years	56 (16.6)	104 (15.7)	
	≥12 years	100 (29.7)	185 (27.9)	
**Occupation n (%)**	White collar occupations	226 (67.1)	422 (63.6)	0.53
	Blue collar occupations	79 (23.4)	167 (25.2)	
	Other (unemployed, student, never had a job)	32 (9.5)	74 (11.2)	
**Current occupation status n (%)**	Working	157 (46.6)	294 (44.3)	0.74
	Retired	133 (39.5)	267 (40.3)	
	Other (unemployed, student, never had a job)	47 (13.9)	102 (15.4)	
**Regular Physical Activity n (%)**	Yes	160 (47.4)	325 (49.0)	0.65
**Chronic medication n (%)**	Yes	115 (34.1)	212 (32.0)	0.49
**Self reported diagnosis n (%)**	Knee OA	38 (11.3)	87 (13.1)	0.40
	Other rheumatic disease	12 (3.6)	26 (3.9)	0.78
	Depression	90 (26.7)	183 (27.6)	0.76
	Other chronic disease	130 (38.6)	240 (36.2)	0.46
**Height (cm) Median (25th–75th percentile)**		161 (155.1-168.3)	160.8 (154.0-168.0)	0.42
**Weight (kg) Median (25th–75th percentile)**		69.8 (61.2-79.1)	69.7 (60.0-79.0)	0.73
**Body mass index (kg/m**^**2**^) **n (%)**	< 25.0 kg/m^2^	118 (35.0)	222 (33.5)	0.81
	25.0-29.9 kg/m^2^	152 (45.1)	314 (45.8)	
	≥30.0 kg/m^2^	67 (19.9)	137 (20.7)	
**Knee Pain (“ever”)**	Yes	133 (39.5)	280 (42.2)	0.40
**BDI score Median (25th–75th percentile)**		6 (3–12)	7 (3–12)	0.59

### Data analysis

Continuous variables were described by mean (standard deviation) for variables with a normal distribution and by median (25th–75th percentile) for skewed distributions. Comparisons were tested using independent t-test for means, Mann–Whitney test for non-parametric distributions and qui-squared for proportions. To estimate the association of pain score and radiographic knee OA with depressive symptoms (BDI ≥ 14) we used odds ratio (OR) and their 95% confidence interval (95% CI) calculated by logistic regression, adjusted for age, BMI and gender. Data analysis was performed using *R*® statistical software, considering a significance level of 5%.

The dimensionality and internal consistency of knee pain questions was assessed by factor analysis for dichotomous variables (latent trait model) and Cronbach’s Alpha, respectively. The ability of the knee pain score to discriminate between patients with or without radiographic KL ≥ 2 OA was summarised by sensitivity, specificity and likelihood ratios (LR = sensitivity/(1 − specificity)) [30,31], stratifying by sex and BDI score.

## Results

**Figure 1:**
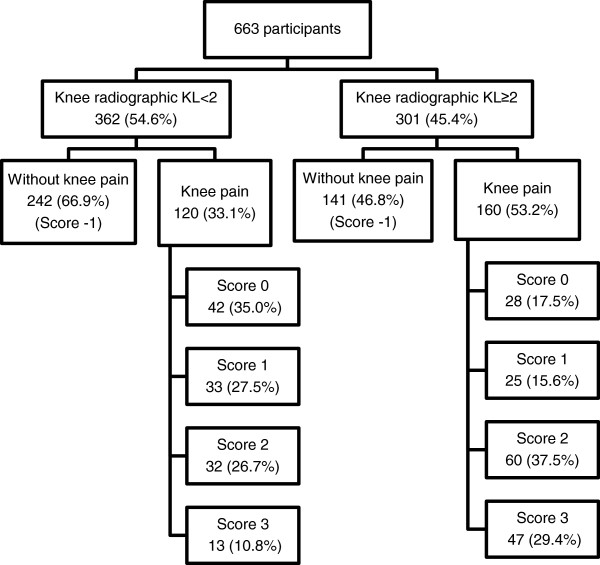
Distribution of participants according to knee pain and radiographic findings.

Factor analysis for knee pain questions (dichotomous variables) identified only one factor and all items showed a factor loading higher than 0.86, and a global Cronbach’s Alpha of 0.66 (Table 2). So we decided to consider these items as a score for knee pain and we classified participants as having no knee pain (score −1), knee pain but no other positive answer (score 0), and score 1, score 2 or score 3, according to the number positive answers. Overall radiographic knee OA (KL ≥ 2) was present in 45.4% of the participants and 42.2% reported “having had knee pain not related with any trauma or injury”. Stratifying by radiographic classification, knee pain was reported by 53.2% of those with radiographic KL ≥ 2 and by 33.2% of those with radiographic KL < 2. Also, the proportion of participants with higher pain scores was larger among those with radiographic KL ≥ 2 (Figure 1).

**Table 2:** Internal consistency and factor analysis for dichotomous variables (latent trait model)

**Question**	**Factor loading**	**Cronbach’s alpha if item deleted**
“In the last year did you have more than 3 knee pain episodes?”	0.91	0.51
“During the last 6 months did knee pain last longer than a week?”	0.86	0.70
“During the last month did you have knee pain?”	0.98	0.46
**Global Cronbach**’**s Alpha**	0.66	

We found that participants with higher pain scores had higher odds of having depressive symptoms. After adjustment for age, BMI and gender and considering participants with no pain (pain score −1) as reference category we found: OR = 1.22 (95% CI 0.53; 2.79) for pain score 0, OR = 1.71 (95% CI 0.77; 3.77) for pain score 1, OR = 3.51 (95% CI 1.92; 6.44) for pain score 2 and OR = 5.64 (95% CI 2.85; 11.16) for pain score 3.

The association between pain score and radiographic knee OA by categories of depressive symptomatology is presented in Table 3. The odds of having radiographic knee OA (KL ≥ 2) was higher in participants with higher scores of pain, both in patients with and without depressive symptoms; however the differences were higher among those with BDI < 14, even after after adjustment for age, BMI and gender.

**Table 3:** Association between radiographic score and knee pain score, according to depressive symptoms

	**Knee pain score**	**Radiographic score**		**Crude**	**Adjusted**
		**KL < 2**	**KL ≥ 2**		
		**n (%)**	**n (%)**	odds ratio*	odds ratio**
	**-1**	222 (70.3)	128 (53.1)	1 (reference category)	1 (reference category)
	**0**	38 (12.0)	24 (10.0)	1.10 (0.63; 1.91)	0.85 (0.46; 1.58)
**BDI < 14**	**1**	27 (8.5)	21 (8.7)	1.35 (0.73; 2.48)	1.03 (0.51; 2.07)
	**2**	24 (7.6)	40 (16.6)	2.89 (1.67; 5.01)	2.28 (1.21; 4.30)
	**3**	5 (1.6)	28 (11.6)	9.71 (3.66; 25.78)	5.37 (1.90; 15.18)
	**-1**	20 (43.5)	13 (21.7)	1 (reference category)	1 (reference category)
	**0**	4 (8.7)	4 (6.7)	1.54 (0.33; 7.26)	1.68 (0.35; 8.18)
**BDI ≥ 14**	**1**	6 (13.0)	4 (6.7)	1.03 (0.24; 4.35)	1.14 (0.25; 5.14)
	**2**	8 (17.4)	20 (33.3)	3.85 (1.31;11.29)	3.60 (1.17; 11.07)
	**3**	8 (17.4)	19 (31.7)	3.65 (1.24; 10.78)	2.73 (0.84; 8.86)

*Crude odds ratio for radiographic OA (KL ≥2); **Adjusted odds ratio for age, body mass index (BMI) and gender.

The sensitivity, specificity and likelihood ratio regarding each knee pain question is described in Table 4. The question with the lowest sensitivity was “During the last 6 months, did knee pain last longer than a week?” (32.9%). The question regarding pain episodes in the last month presented the highest sensitivity value (72.8%). In general, small likelihood ratios were obtained regarding the pre and post-test probability of having radiographic OA, showing the low ability of these questions to identify participants with knee radiographic OA.

**Table 4:** Diagnostic accuracy of each knee pain question to identify participants with knee radiographic KL ≥ 2, total and by sex

**Question**	**All participants**				**Female**				**Male**			
	**n (%)**	**Sensitivity%**	**1-Specificity**	**Like**-**lihood Ratio**	**n (%)**	**Sensitivity%**	**1-Specificity**	**Like**-**lihood Ratio**	**n (%)**	**Sensitivity%**	**1-Specificity%**	**Like-lihood Ratio**
“**Have you ever had knee pain not related with any trauma or injury?” (yes)**	280 (42.2)	53.2	33.2	1.60	192 (51.8)	63.9	41.6	1.54	88 (30.1)	39.4	22.5	1.75
“**In the last year did you have more than 3 knee pain episodes?”(yes)**	172 (61.0)	71.4	47.0	1.52	131 (68.2)	78.7	54.8	1.44	41 (45.6)	56.6	29.7	1.90
“**During the last 6 months did knee pain last longer than a week?”(yes)**	72 (25.5)	32.9	15.7	2.10	50(26.0)	33.3	16.7	2.0	22(24.4)	32.1	13.5	2.37
“**During the last month did you have knee pain?” (yes)**	178 (62.9)	72.8	49.6	1.47	131 (67.9)	76.1	57.1	1.33	47 (52.2)	66.0	32.4	2.04

Considering the pain score (Table 5), score −1 (participants that reported no pain) showed a high sensitivity to identify patients with knee OA (46.8%), which probably reflects the high number of participants with knee radiographic KL ≥ 2 but without pain; however this score also presented a very low specificity (33.1%).

**Table 5:** Diagnostic accuracy of knee pain scores to identify participants with knee radiographic KL ≥ 2, total and by sex

	**All participants**				**Female**				**Male**			
	**n (%)**	**Sensitivity%**	**1**-**Specificity%**	**Likelihood Ratio**	**n (%)**	**Sensitivity%**	**1-Specificity%**	**Likelihood Ratio**	**n (%)**	**Sensitivity**	**1-Specificity**	**Likelihood Ratio**
**Score**												
−**1**	383 (57.8)	46.8	66.9	0.70	179 (48.2)	36.1	58.4	0.62	204 (69.9)	60.6	77.5	0.78
**0**	70 (10.6)	9.3	11.6	0.80	37 (10.0)	8.9	10.9	0.82	33 (11.3)	9.8	12.5	0.79
**1**	58 (8.7)	8.3	9.1	0.91	42 (11.3)	9.5	12.9	0.74	16 (5.5)	6.8	4.3	1.56
**2**	92 (13.9)	19.9	8.8	2.26	69 (18.6)	25.4	12.9	1.98	23 (7.9)	12.9	3.8	3.43
**3**	60 (9.0)	15.6	3.5	4.35	44 (11.9)	20.1	5.0	4.06	16 (5.5)	9.8	1.9	5.25

Among those that reported at least one positive answer on the pain questionnaire (scores from 0 to 3) the ability of knee pain to identify patients with radiographic knee OA increased with increased scores. When we used only the “have you ever had knee pain” question (score 0) or even with score 1 (“have you ever had knee pain” and another positive question) a very low sensitivity was reached (9.3% and 8.3%, respectively) and we obtained a low discrimination ability (positive likelihood ratio of 0.80 and 0.91). Scores 2 and 3 presented a high specificity (>90%) but a low sensitivity (<20%), but nevertheless this was twice as high as the sensitivity obtained with the score 1 and 2. Based on the positive likelihood ratio, the pos-test probability of having a knee radiographic KL ≥ 2 for those who scored 2 was twice that of the pre-test probability and this increased to 4.35 when participants scored 3. Similar results were found by sex. However, additional positive answers in males contributed to higher likelihood ratios than in females (Table 5).

To analyze the role of depressive symptoms in the discrimination ability of knee pain to identify individuals with radiographic OA, we decided not to stratify by sex in order to have enough power. The prevalence of depression (BDI > 14) was 19.9% among participants with radiographic KL ≥ 2 and 12.6% in those with radiographic KL < 2 (*p* = 0.01). Among participants with BDI ≤ 14 additional positive answers (increased knee pain score) allowed an increase in the positive likelihood ratio: those with score 2 had twice the probability of having radiographic KL ≥ 2 than before the questions and this increased to 7.34 for those who scored 3. In the presence of depressive symptoms BDI > 14 the ability of these questions to identify participants with radiographic knee OA became lower, with a positive likelihood ratio of 1.92 for those who scored 2 and 1.82 for those who scored 3 (Table 6).

**Table 6:** Diagnostic accuracy of knee pain scores to identify patients with and without radiographic knee OA, according to depressive symptoms

	**BDI** ≤ **14**				**BDI > 14**			
	**n (%)**	**Sensitivity%**	**1**-**Specificity%**	**Likelihood ratio**	**n (%)**	**Sensitivity%**	**1**-**Specificity%**	**Likelihood ratio**
**Score**								
−**1**	350 (62.8)	53.1	70.3	0.76	33 (31.1)	21.7	43.5	0.50
**0**	62 (11.1)	10.0	12.0	0.83	8 (7.5)	6.7	8.7	0.77
**1**	48 (8.6)	8.7	8.5	1.02	10 (9.4)	6.7	13.0	0.51
**2**	64 (11.5)	16.6	7.6	2.19	28 (26.4)	33.3	17.4	1.92
**3**	33 (5.9)	11.6	1.6	7.34	27 (25.5)	31.7	17.4	1.82

## Discussion

In our study, a high proportion of participants had radiographic findings but did not report knee pain (21.3%). This is in accordance with previous data [1,32-34]. Pain is the most frequent complaint in OA and frequently the first reason for consulting a physician [12]. It is an unspecific symptom and its expression may be associated with other conditions than OA [34,35], making pain assessment a relevant but difficult issue [36]. We found that participants with higher pain scores had higher odds of having depressive symptoms. Additionally, a higher association of knee pain with radiographic knee OA was found in participants without depressive symptoms. The probability of a participant having radiographic KL ≥ 2 rose substantially with the number of positive answers to knee pain questions (increased pain scores). This is more obvious in score 2 and 3 where the higher likelihood ratios were found. These results reinforce our idea that using a score was better than using each separate question and they were in accordance with previous studies showing a lower ability of individual questions on knee pain for the diagnosis of OA [11].

Our results were consistent in females and males, but positive answers in males contributed to higher likelihood ratios than in females. This may be explained by sex differences in pain perception, evaluation, and reporting, with females reporting pain more frequently than males, which may make these symptoms more unspecific in females [37-39].

Since, in general, our data showed similar results by sex, and in order to improve the statistical power, we decided to analyze the role of depressive symptoms not stratifying by sex. Among participants with BDI ≤ 14 the likelihood ratio to identify patients with radiographic knee OA increased with increased pain scores reaching 7.34 when participants responded positively to all pain questions (score 3). In the presence of depressive symptoms (BDI > 14) our score became unable to identify participants with radiographic knee OA since the likelihood ratios ranged from 0.77 for those with one positive answer (score 0) to 1.82 for those with all positive answers (score 3). These results are in accordance with other studies that show that depressive symptoms can change pain perception and contribute to pain over-expression [20,35,40]. Thus, in the presence of depressive symptoms, pain becomes more unspecific so the ability of pain complains to allow the identification of patients with radiographic OA is lower.

It is important to highlight that our data was from a population-based study and participants had low depressive symptomatology [median of 7 (25th–75th percentile: 3–12)]. It can be argued that in populations with more prevalent depressive symptoms, such as older populations or those with higher co-morbidities, knee pain can even have a lower discrimination ability to identify participants with OA.

On the other hand, among participants with depressive symptoms, pain questions could be useful to identify those without disease, since negative answers (reporting no pain) allowed the best specificity (57%) and a positive likelihood ratio of 0.50.

Considering all participants, the score −1, corresponding to participants that reported no pain, was unable to efficiently discriminate participants according to radiographic OA findings (it presented a high sensitivity, but a very low specificity and the lowest likelihood ratio). So, reporting “no knee pain” was not enough to accurately identify participants without radiographic OA. However, stratified analysis by depressive symptoms, showed that among participants with depressive symptoms the specificity of score −1 rose to almost 60%, showing that in this group of participants reporting “no knee pain” may be a better marker to identify participants without radiographic OA.

Pain related to OA is associated with an increased risk of worse psychological outcomes, including anxiety, depression and helplessness [15,18,41]. Since our data is based on a cross-sectional study, it is impossible to understand if depressive symptoms are responsible for OA pain manifestations or whether they are caused by OA pain. However, this was not the purpose of our study. We tried to understand if the presence of depressive symptoms, regardless of their causes, may influence knee pain expression and its ability to identify patients with relevant radiographic OA findings.

We tried to minimize the problem of pain assessment using questions with different time frames. Nevertheless, the ability of knee pain to identify participants with radiographic OA would probably improve with a higher number of questions; however, we tried to use a small number of easy questions, which could be easily used in a clinical setting or in population based studies.

One limitation of our study was the inability to explore other common features of OA such as stiffness, loss of joint mobility, swelling, tenderness, joint deformity and muscle weakness. However, pain is thought to be one of the aspects that is more correlated with radiographic symptomatic changes [10] and is also highly associated to the other OA signs and symptoms, namely disability [32,42]. So we could expect a similar effect of depressive symptoms on those signs and symptoms.

In our study radiographic OA was defined as *Kellgren Lawrence* score of 2–4, and some reliability and validity limitations of this grading scale can be discuss [43]. Our radiographs were scored only by one experienced reader that was unaware of the participants’ clinical data. We used a cut off point ≥ 2 to define radiographic OA which is also a controversial aspect [26,27]. Since differences would be expected in the relation between pain and radiographic changes according to disease severity [21], we performed the same analyses using KL ≥ 3 as cut off point and we found a slight improvement in the ability of knee pain to identify participants with radiographic OA, but those differences disappeared among those with depressive symptoms (data not shown).

Although our study was developed from a population-based study, we lost almost 33% of the participants for our research, which could determine a selection bias. Nevertheless, non-participants were quite similar to the studied population although younger. Thus, despite some limitations, our results support the idea that psychological status deserves greater clinical and research attention in OA, since the presence of depressive symptoms impairs the ability of pain complaints to identify potential OA patients or to correctly manage the disease in those already diagnosed. The evaluation and treatment of depressive symptoms should be an integrative part of OA patient management, in order to improve it and to ensure that knee pain expression can be correctly understood.

## Conclusions

Our study highlights the importance of a more comprehensive understanding of pain complaints to improve our ability to identify individuals with OA and to apply rational treatment strategies.

Knee pain was associated both with depressive symptoms and radiographic OA. Single questions on knee pain symptoms do not allow the identification of patients with radiographic OA, but better discrimination ability was found using a score of four questions, with higher scores showing the higher discrimination ability in both sexes. However, the presence of depressive symptoms impairs the ability of knee pain complaints in OA diagnosis and management.

## Competing interests

The authors declare that they have no competing interests.

## Authors’ contributions

DP and ER participated in the study conception, design and drafting. RAS was involved in the data collection and study design. MS performed the statistical analysis and participated in data interpretation. JB and HB were involved in revising it critically for important intellectual content. All authors read and approved the final manuscript.

## Pre-publication history

The pre-publication history for this paper can be accessed here:

http://www.biomedcentral.com/1471-2474/14/214/prepub
